# Supplementary material for: Auditory Steady-State Responses in Schizophrenia: An Updated Meta-Analysis
Source: Brain Sci. 2023 Dec 16;13(12):1722. doi: 10.3390/brainsci13121722 (PMC10741772; doi:10.3390/brainsci13121722)
Supplement: Supplementary file 1 [file brainsci-13-01722-s001.zip › brainsci-2754628-supplementary.pdf]

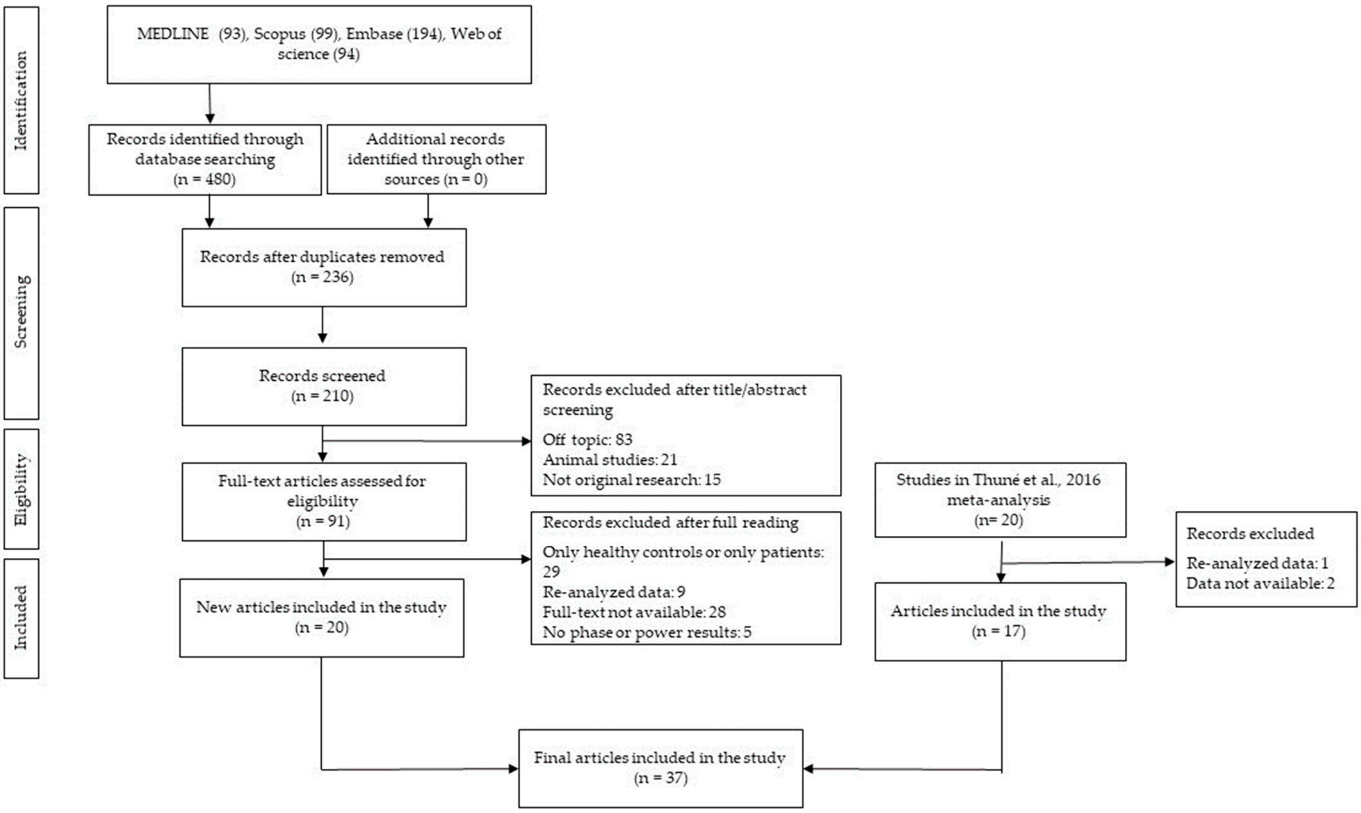

Supplementary Figure S1. PRISMA Flow Diagram depicting the systematic selection process of studies for inclusion in our analysis.

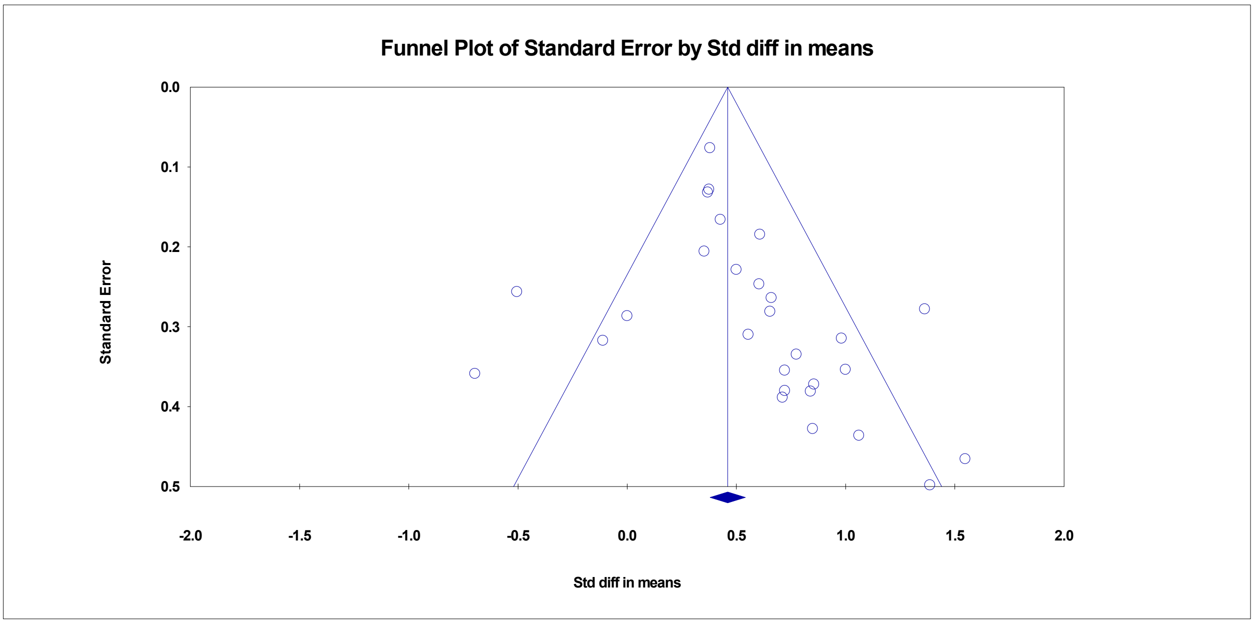

Supplementary Figure S2-Funnel Plot of Standard Error (vertical axis) versus Standardized Difference in Means (horizontal axis) for the power component of 40 Hz Auditory Steady-State Responses: The scatter distribution assesses potential publication bias and methodological variance.

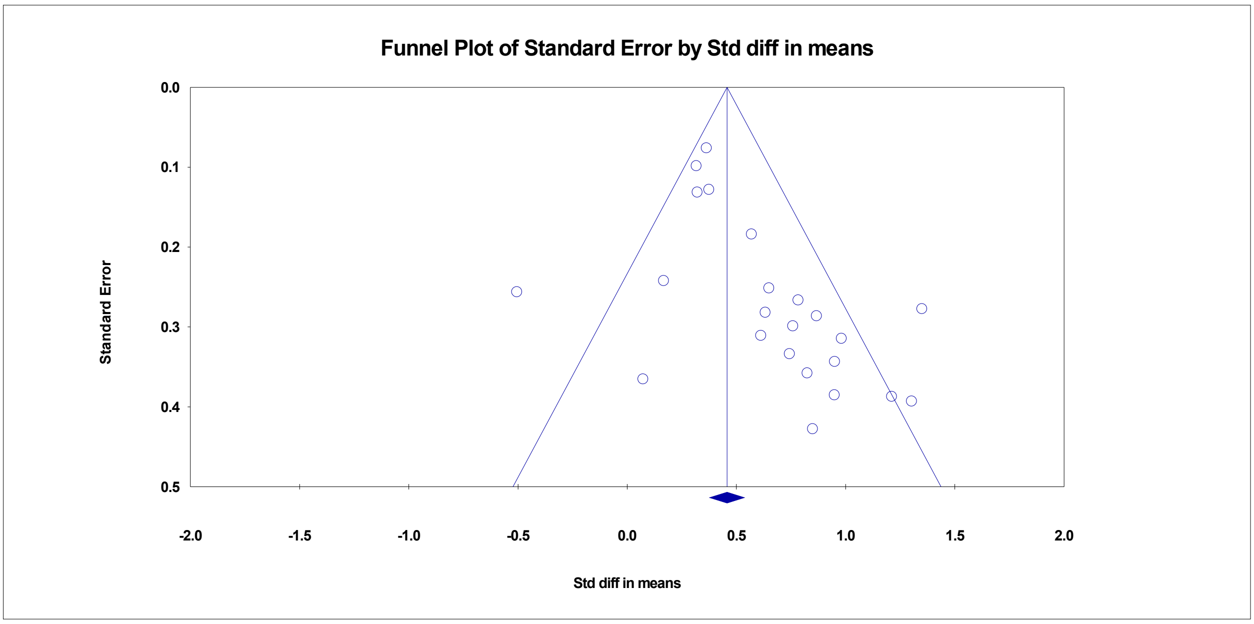

Supplementary Figure S3. Funnel Plot of Standard Error (vertical axis) versus Standardized Difference in Means (horizontal axis) for the phase component of 40 Hz Auditory Steady-State Responses.

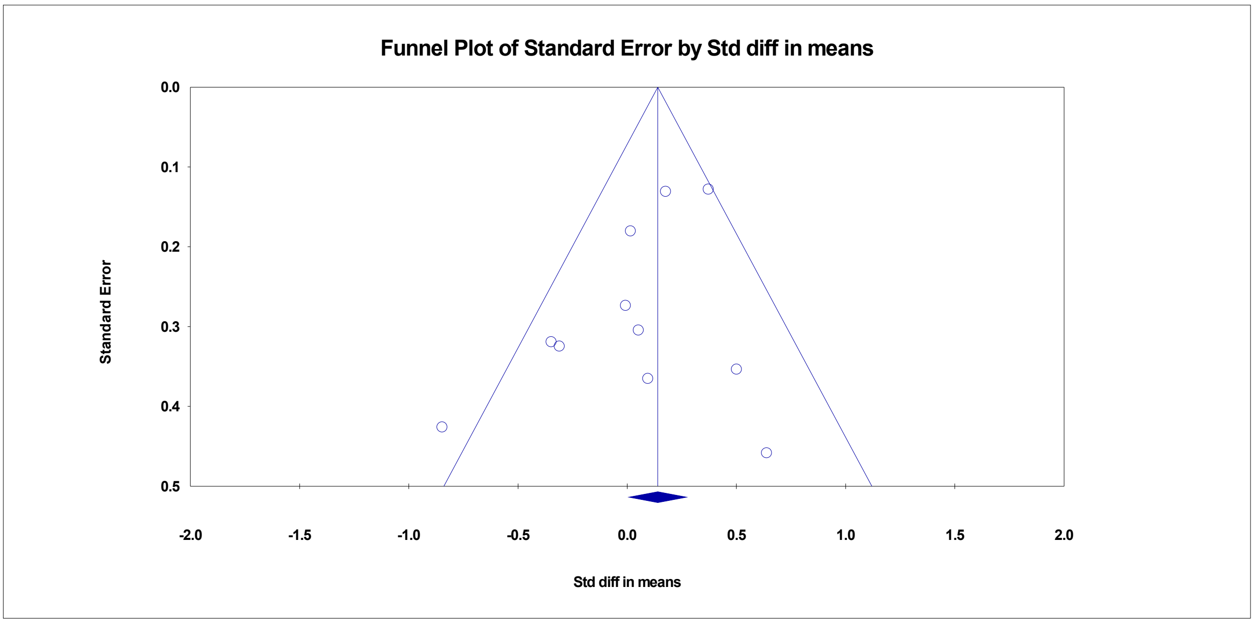

Supplementary Figure S4. Funnel Plot of Standard Error (vertical axis) versus Standardized Difference in Means (horizontal axis) for studies on the power component of beta oscillations in Auditory Steady-State Responses.

Supplementary Table S1. Participants characteristics across studies.

Authors	Patient group	N controls	N patients*	% males patients*	Mean age (y) controls	Mean age (y) patients*	Illness duration (y): Mean (SD)*	CPZ (mg/day): Mean (SD)*	Positive symptoms** Mean (SD)*	Negative symptoms** Mean (SD)*
Kwon et al., 1999 [32]	SCZ	15	15	100	44.6	43.3	21.1 (7.1)	440.1 (261.6)	17.1 (6.9)	18.1 (7)
Hong et al., 2004 [33]	SCZ, AR	17	SCZ = 24<br>AR = 11	SCZ = 58<br>AR = 55	41.1	SCZ = 39.7<br>AR = 44.4		348.7 (150.45)		
Spencer et al., 2008 [34]	FEP	33	FEP = 16	FEP = 58	27.5	FEP = 25.5		FEP = 338 (196)	FEP = 20.9 (4.5)	FEP = 17.8 (5.5)
Vierling-claasen et al., 2008 [35]	SCZ	12	12	100			26.2 (8.2)			
Teale et al., 2008 [36]	SCZ	15	15	87	34.8	37.9	12.6 (7.6)	499		
Wilson et al., 2008 [37]	FEP	10	FEP = 10	FEP = 70	15.8	FEP = 14.6	3.44 (2.97)			
Spencer et al., 2009 [38]	SCZ	16	18	100	44.4	39.8	13.7 (8)	450 (306)	13.5 (12.3)	11.8 (8.8)
Hamm et al., 2011 [39]	SCZ	18	18	89	39.7	40.7	18.2	207.2	19.5	13.9
Tsuchimoto et al., 2011 [40]	SCZ	22	17	35	37	35.6	13.5 (7.5)	541 (367)	13.1 (12.6)	15.8 (8.8)
Hamm et al., 2012 [41]	SCZ	16	17	65	39.5	41.5	18.2 (7.88)	355 (245)		
Kömek et al., 2012 [42]	SCZ	12	12	58	31.4	30.3				
Rass et al., 2012 [43]	SCZ, AR	56	SCZ = 42<br>AR = 35	SCZ = 55<br>FEP = 37	38.75	SCZ = 36.9<br>AR = 36				
Kirihara et al., 2012 [44]	SCZ	188	234	78	43.9	44.5	22.7 (9.9)		13.4 (12.2)	11.7 (8.6)
Roach et al., 2013 [45]	SCZ	25	28	75	36.1	39.3			15.4 (5)	14.3 (5.2)
Edgar et al., 2014 [46]	SCZ	29	39	85	37.9	40.9			17.8	15.8
Hirano et al., 2015 [47]	SCZ	24	24	83	44.1	46	21.1 (9.7)	426.39 (444.03)		
Hamm et al., 2015 [20]	SCZ	18	18	50	40.8	45.6		207	13.7	14.8
Griskova-Bulanova, 2016 [48]	SCZ	25	26	65	35.2	39.5		424.1 (358.82)	21.62 (6.28)	20.23(7.24)
Alegre, 2017 [49]	SCZ, FEP	13	SCZ = 17<br>FEP = 11	SCZ = 41<br>FEP = 64	27.6	SCZ = 38.5<br>FEP = 32.6	2 (10.29)	SCZ = 562.5 (380.7)<br>FEP = 0		
Light, 2017 [50]	SCZ	14	18	61	26.6	37.2	21.18	586.73 (122.43)	17.6 (1.1)	17.4 (1.9)
Edgar, 2018 [51]	SCZ	55	41	80	39.6	40.3			17.1	15.1

Griskova-Bulanova, 2018 [52]	SCZ	20	26	100	38	42	17 (12)	692.23 (310.38)	21.8 (5.9)	28.8 (5.6)
Koshiyama, 2018 [31]	FEP, AR	30	FEP = 34<br>AR = 28	FEP = 100<br>AR = 81	22	FEP = 23.9<br>AR = 21.4	1.15 (1.04)	FEP = 529 (385)	FEP = 14.7 (5.3)	FEP = 18.7 (5.7)
Puvvada, 2018 [53]	SCZ, AR	108	SCZ = 128<br>AR = 55	SCZ = 67<br>AR = 31	37.9	SCZ = 37.8<br>AR = 46.6				
Sun, 2018 [54]	SCZ	30	24	54	34.2	33	9.06 (9.18)	514.7 (246.4)	20.6 (6.3)	23.9 (6.8)
Wang, 2018 [55]	FEP	28	FEP = 33	FEP = 42	26.1	FEP = 24.7	0.41 (0.41)	FEP = 193.36 (129.16)	FEP = 20.7 (3.7)	FEP = 15.9 (4.9)
Zhou, 2018 [56]	SCZ	75	52	21	38.9	42.7	19 (8.3)	590.2 (560.7)	17 (6.7)	18 (8.9)
Bartolomeo, 2019 [57]	FEP	19	FEP = 34	FEP = 71	22.9	FEP = 22	2.01 (1.33)	FEP = 139.6 (173)	FEP = 16.9 (6.5)	FEP = 12.7 (5.6)
Kim, 2019 [58]	SCZ	30	33	48	43.3	42.2	13.57 (8.9)	495.06 (558.53)	13.1 (6.6)	16.5 (6.2)
Parker, 2019 [59]	SCZ	137	113	55	41.7	39		580 (851)	18.3 (6.6)	18.8 (7.2)
Lepock, 2020 [60]	AR	22	AR = 36	AR = 67	21.7	AR = 21.3				
Murphy, 2020 [61]	SCZ, FEP	17	SCZ = 16<br>FEP = 12	SCZ = 81<br>FEP = 100	28.8	SCZ = 33.6<br>FEP = 27.5	SCZ = 11.5 (6.4)<br>FEP = 2.6 (5.8)		SCZ = 13.4 (12.1)<br>FEP = 12.5 (11.8)	SCZ = 10.0 (8.6)<br>FEP = 9.3 (8.5)
Grent-'t-Jong, 2021 [62]	FEP, AR	49	FEP = 33<br>AR = 116	FEP = 67<br>AR = 29	23	FEP = 24<br>AR = 22			FEP = 20 (8)	FEP = 16 (9.2)
Koshiyama, 2021 [63]	SCZ	294	433	69	43.7	45.5	23.9 (10.9)			11.5 (8.7)
Coffman, 2022 [64]	FEP	32	FEP = 25	FEP = 84	24	FEP = 24		FEP = 199.2 (154.9)	FEP = 20.1 (5)	FEP = 18.7 (5.9)
Du, 2023 [65]	SCZ	85	66	67	35.6	33.9		573 (548)		
Ogyu, 2023 [66]	SCZ	25	54	52	40.2	41	17.3 (12)	681.75 (461.75)	18.25 (3.65)	20.15 (5.95)

*: values for SCZ group when not specified

**: as measured with the Positive and Negative Syndrome Scale

SCZ = chronic schizophrenia, FEP = First episode psychosis, AR = at-risk, y = in years, SD = Standard Deviation, CPZ = Chlorpromazine dosage equivalent
